# Propofol Versus Sevoflurane General Anaesthesia for Selective Impairment of Attention Networks After Gynaecological Surgery in Middle-Aged Women: A Randomised Controlled Trial

**DOI:** 10.3389/fpsyt.2022.917766

**Published:** 2022-07-14

**Authors:** Chen Chen, Yuxue Wang, Jin Rao, Weixiang Tang, Weiwei Wu, Yuanhai Li, Guanghong Xu, Weiwei Zhong

**Affiliations:** ^1^Department of Anaesthesiology, The First Affiliated Hospital of Anhui Medical University, Hefei, China; ^2^Department of Anaesthesiology, Fourth Affiliated Hospital of Anhui Medical University, Hefei, China

**Keywords:** attention network, propofol, sevoflurane, postoperative cognitive dysfunction, postoperative neurocognitive disorder

## Abstract

**Purpose:**

Attention is an essential component of cognitive function that may be impaired after surgery with anaesthesia. Propofol intravenous anaesthesia and sevoflurane inhalational anaesthesia are frequently used in gynaecological surgery. However, which type of anaesthetic has fewer cognitive effects postoperatively remains unclear. We compared the differences in attention network impairment after surgery in women receiving propofol versus sevoflurane general anaesthesia.

**Patients and Methods:**

Eighty-three patients with gynaecological diseases who were 40–60 years of age were involved in the study. All patients underwent elective gynaecological surgery under either total intravenous anaesthesia or sevoflurane inhalational anaesthesia, depending on randomisation. The efficiencies of the three attention networks were captured using the attention network test preoperatively and on the 1st and 5th postoperative days.

**Results:**

Both groups of patients showed differences in impairments on the 1st and 5th postoperative days. Pairwise comparisons indicated that the alerting and orienting networks of patients in the propofol group were impaired to a greater extent than those of patients in the sevoflurane group on the 1st postoperative day, while the executive control network was impaired to a lesser extent. On the 5th postoperative day, the alerting networks of both groups recovered to the baseline level. Patients in the propofol group still showed impairment of the orienting network, while patients in the sevoflurane group recovered to baseline. For the executive control network, patients in the sevoflurane group still exhibited more severe impairment than those in the propofol group.

**Conclusion:**

In middle-aged women, propofol impaired orienting and alerting networks more than sevoflurane, while sevoflurane showed more residual impairment of the executive control network.

## Highlights

- We studied postoperative cognitive impairment in middle-aged women after different types of anaesthesia.
- Sevoflurane inhalation and propofol intravenous anaesthesia induced different degrees of impairment and recovery in regard to the three aspects of the attention network.
- We speculate that sevoflurane and propofol act on different regions of the brain and thus speculate that they act on the main target areas of the brain.

## Introduction

Postoperative cognitive dysfunction (POCD) is a common complication of the central nervous system after surgery and anaesthesia (1) that can affect patients in different age groups, with incidences ranging from 7 to 80%, especially in women (2–5). POCD can lead to increased complications and mortality, delayed rehabilitation, prolonged hospital stay, and poor quality of life of patients after discharge. Moreover, the 5-year mortality rate caused by POCD is estimated to be 70% (6) which places a heavy medical burden on patients and society.

Postoperative cognitive dysfunction shows various cognitive deficits, including a decline in memory, attention, and psychomotor speed (7). Attention is the general term of various psychological phenomena, which represents the ability to choose from stimuli, reactions, memories and thoughts and ignores any irrelevant information in the process (8). It is generally believed that patients with cognitive disorders such as Alzheimer’s disease (AD) and schizophrenia have attention deficits (9, 10). According to cognitive neuroscience models of attention, there are three attention networks: alerting, orienting and executive control (11). These networks are responsible for different functions in attention process (12) and the efficiency of these three networks can be captured using attention network testing (ANT), which was designed by Fan et al. (13). ANT measures the activities of all three networks at the same time and evaluates their relationship based on the flanking and exogenous clue paradigm. Given the facts above, we can use ANT to study the relationship between anaesthesia and POCD by detecting the attention network.

Although the aetiology and pathogenesis of POCD are uncertain and multifactorial (7), a causative link to general anaesthesia is increasingly recognised (1, 4). At present, most patients need general anaesthesia during surgery, and many studies have shown that general anaesthesia can lead to a decline in the postoperative cognitive function of patients (2, 14). Because anaesthetic choice can be effectively managed by anaesthesiologists, it is important to determine the role of anaesthetic choice in POCD. Propofol and sevoflurane are common general anaesthetics used in clinical anaesthesia, and animal experiments have also shown that propofol and sevoflurane can cause the occurrence of POCD (15, 16). The choice of anaesthetic may affect cognitive outcome after the operation, but the results from clinical research have always been contradictory. For example, Zhang and others showed that sevoflurane inhalation anaesthesia may have a higher risk of POCD than propofol intravenous anaesthesia (17). Schoen and others showed that sevoflurane anaesthesia was associated with better short-term cognitive function after surgery than propofol anaesthesia (18). It is still unclear which type of general anaesthetic has less effect on cognitive function (19). Moreover, whether intravenous and inhaled anaesthetics are associated with different risks of attention network impairment has not been reported.

Gender differences in cognitive functioning have been consistently reported in some cognitive tasks, with varying effect sizes (20). For example, men perform better on spatial memory, while women excel at verbal memory (21). Women are more likely to show mental and nervous system diseases with cognitive impairment, such as depression and AD (21, 22).

The purpose of this study was to compare the types of cognitive changes and differences in attention network impairment among women receiving propofol intravenous anaesthesia versus sevoflurane inhalation anaesthesia and to observe the recovery trajectories of attention network impairment.

## Materials and Methods

### Trial Registry and Ethical Approval

This trial was approved by the Chinese Clinical Trial Registry (ChiCTR-1900024311). Each patient provided written informed consent before participating in the study, and all procedures were conducted according to the Declaration of Helsinki. Ethical approval was approved by the Ethics Committee of Anhui Medical University in Hefei, Anhui, China, in July 2013 (Ethics Committee No. 20130406, Chairperson, Professor Run-ling Wang).

### Participants and Exclusion Criteria

A total of 120 patients aged 40–60 with gynaecological diseases were involved in the study. All patients underwent elective gynaecological surgery (hysterectomy) with either propofol intravenous anaesthesia (PROPOFOL) or sevoflurane (SEVO) inhalational anaesthesia at the First Affiliated Hospital of Anhui Medical University from July 2019 to December 2019. The exclusion criteria were as follows: an ASA status of III or IV; history of respiratory or circulatory system diseases; history of neurological and psychiatric disorders; a Mini-Mental State Examination score ≤23; inadequate postoperative analgesia with a visual analogue scale (VAS) ≥ 3; or unwillingness to comply with or difficulty in understanding the protocol or procedures at any time during the trial.

### Randomisation

A total of 120 patients were randomly divided into the PROPOFOL group and the SEVO group at a 1:1 ratio by a computer programme. The patients and research staff who observed and followed up with the patients after surgery were blinded to the group assignment.

### Study Timeline

Baseline characteristics, including age, education level, weight, preoperative Mini-Mental State Examination score, and underlying diseases were assessed and recorded 1 day preoperatively. Duration of surgery and Sufentanil dosage were recorded during surgery. All participants completed the ANT test 1 day preoperatively and on the 1st and 5th days postoperatively. The different types of changes in recovery trajectories related to attention networks were compared between those receiving PROPOFOL versus SEVO anaesthesia.

### Anaesthesia

After arrival in the operating room, all patients underwent intravenous access without any premedication and commenced receiving 4 l/min oxygen through a face mask. Patient monitoring included heart rate, pulse oximetry, ECG, end-tidal concentrations of carbon dioxide, non-invasive blood pressure and bispectral indices (BIS).

For the PROPOFOL group, after intravenous preloading, all patients received propofol (1.5–2.5 mg/kg), sufentanil (0.3–0.5 μg/kg), and cis-atracurium besylate (0.2 mg/kg) during anaesthesia induction. A laryngeal mask airway was inserted 3–5 min after induction. To maintain the appropriate depth of anaesthesia, propofol (1.5–4 μg/ml) and remifentanil (0.1–0.4 μg/kg/min) were given by continuous intravenous infusion, and cisatracurium besylate was used intermittently.

For the SEVO group, all patients were given 8% sevoflurane [fresh gas flow (FGF) 6 l/min], sufentanil (0.3–0.5 μg/kg), and cisatracurium besylate (0.2 mg/kg) when induced, and then sevoflurane was adjusted to 3–4% (FGF 1–2 l/min) after loss of consciousness. After endotracheal intubation, sevoflurane was kept between 0.7 and 1.5 minimum alveolar concentration (MAC). Remifentanil (0.1–0.4 μg/kg/min, continuous intravenous infusion) and cisatracurium besylate were used for analgesia and muscle relaxation, respectively.

Bispectral indices was monitored and set to the target range of 40–60 in both groups by adjusting either the flow of sevoflurane or the target blood concentration of propofol.

Complications were managed according to the study protocol of our department. An intravenous bolus of 6 mg ephedrine was used for hypotension (systolic blood pressure < 90 mmHg or 20% less than baseline); an intravenous bolus of 0.3 mg atropine was used for bradycardia (heart rate < 50 beats/min).

At the end of surgery, patient-controlled intravenous analgesia was administered with flurbiprofen (1 mg/ml) and sufentanil (0.02–0.03 μg/kg/ml), which was set at a background infusion rate of 2 ml/h, and a pump set at 0.5-ml boluses with a locking time of 15 min for effective postoperative analgesia. The VAS score was used to assess postoperative pain after surgery.

### ANT Procedure

We used E-Prime (version 1.1; Psychology Software Tools, Pittsburgh, PA, United States) to conduct the attention network test, as we did in our previous research. The test began with an instruction that stressed the importance of rapid and accurate responses and that was followed by 24 practice trials for patients to become familiar with the test. The patients viewed the stimuli on a computer, and accuracy and reaction times were calculated and recorded when they pressed two response buttons (the “←” key and the “→” key).

The ANT can be divided into the following procedures. First, the participants saw a fixation cross (“+”) in the central area that did not disappear until the trials were terminated. Then, at approximately 400–1,600 ms after the appearance of the fixation cross, the patient noticed a cue (“*”) lasting for 100 ms. After 500 ms, the participants observed a target above or below the fixation cross that remained on the screen until they gave a response or until 1,700 ms later. At the end of the trial, the fixation cross disappeared. Each trial lasted approximately 4,000 ms.

**FIGURE 1:**
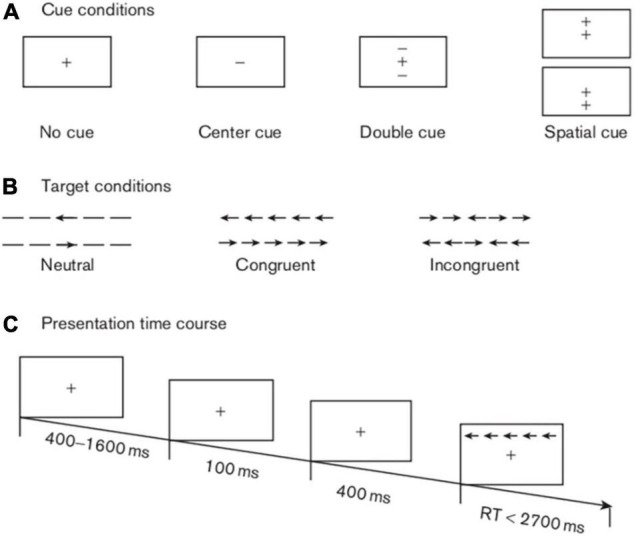
Schematic of the attention network test. This schematic shows the experimental procedure, the four cue conditions, and the three types of target stimuli.

Four kinds of cue conditions existed in this test: (i) no cue, in which the participants did not receive any clue; (ii) a centre cue, in which the participants caught sight of an asterisk in the middle of the screen; (iii) double cues, in which the participants saw two asterisks concurrently above and below the fixation across; and (iv) a spatial cue, in which the participants saw an asterisk either above or below the target. The test demonstrated three types of target stimuli. The first type was “neutral”, in which a single arrow appeared above or below the fixation cross. The second type was “congruent,” in which five arrows emerged in the same direction concurrently with the middle arrow serving as the target. The third type was “incongruent,” in which five arrows were presented simultaneously above or below the fixation cross with the central target arrow in the opposite direction from the other four arrows (Figure 1).

As stated above, in this experiment, there were four cues (no cue, centre cue, double cues, and spatial cue), three targets (neutral, congruent, and incongruent) and three sessions (baseline, 1 day postoperative, and 5 days postoperative). Our study mainly explored the efficiencies of three attention networks, as evaluated by the extent to which the cues affected the response times (RTs), as previously described (13, 23). Specifically, the first attention network was the alerting network, with an effect score equal to the no cue RT minus the double cues RT; the higher the score was, the stronger the alerting function. The second attention network was the orienting network, with an effect score equivalent to the centre cue RT minus the spatial cue RT; the higher the score was, the better the orienting function. The last attention network was the executive control network, in which the effect score stemmed from the incongruent target RT minus the congruent target RT; the lower the score was, the more efficient the execution function.

### Statistical Analyses

Demographics, education level, Mini-Mental State Examination scores, ASA class and intraoperative data, which included the operation duration and drug dose, were compared between the two groups. The ANT was administered at baseline and on days 1 and 5 postoperatively.

All analyses were conducted using SPSS software [SPSS software (version 23.0; SPSS Inc., Chicago, IL, United States)]. All quantitative data were tested for normality by using the Shapiro–Wilk test. Normally distributed variables were summarised using the mean and standard deviation and compared using a t test for demographic data and repeated-measures analysis of variance (RMANOVA) for attention network efficiency. The non-parametric data were analysed with the Mann–Whitney U test. All categorical data were tested using Pearson’s chi-squared test or Fisher’s exact test. A *p* value < 0.05 (two sided) was considered to indicate statistical significance.

## Results

A total of 120 patients were screened for the study, among whom 19 declined to participate in the ANT, 12 were lost due to early discharge, and 6 had a VAS score above 3 after analgesia. The remaining 83 patients (43 patients in the propofol intravenous anaesthesia group and 40 patients in the sevoflurane inhalational anaesthesia group) participated in and completed the study. Subjects in the propofol intravenous anaesthesia and sevoflurane inhalational anaesthesia groups had similar baseline characteristics, including age, education level, weight, preoperative Mini-Mental State Examination score, duration of surgery, and underlying diseases (Table 1).

**TABLE 1:** Demographic and baseline characteristics of patients.

Characteristic	Propofol group (*n* = 43)	Sevoflurane group (*n* = 40)	*P-value*
Age, mean ± SD, years	48.07 ± 4.37	48.93 ± 3.88	0.350
Weight, mean ± SD, kg	60.26 ± 6.90	61.56 ± 8.72	0.450
Diabetes, *n* (%)	1 (2.33)	1 (2.50)	0.959
Hypertension, n (%)	6 (13.95)	4 (10.00)	0.580
Anaemia, *n* (%)	21 (48.84)	17 (42.50)	0.563
Education level			0.587
Illiteracy, *n* (%)	5 (11.63)	4 (10.00)	
Primary school, *n* (%)	6 (13.95)	9 (22.50)	
Junior school, *n* (%)	18 (41.86)	11 (27.50)	
High school, *n* (%)	10 (23.26)	13 (32.50)	
University and above, *n* (%)	4 (9.30)	3 (7.50)	
Duration of surgery, min	123.72 ± 38.54	114.88 ± 34.90	0.278
Sufentanil dosage, μg	43.02 ± 7.29	41.19 ± 5.19	0.193
MMSE	28.09 ± 1.09	28.15 ± 1.17	0.818

*Data are presented as the mean ± SD or number (%). No significant differences were found between the two groups with respect to these characteristics. MMSE, Mini-Mental State Examination.*

### Accuracy

In this study, accuracy refers to the percentage of correct responses in all attention networks, including the alerting, orienting and executive control networks. The accuracy rates were similar across the testing time points and between the PROPOFOL and SEVO groups (Table 2).

**TABLE 2:** Accuracy of patients (x ± s).

	Baseline	1st postoperative day	5th postoperative day
Propofol group (%)	94.30 ± 3.96	91.93 ± 6.90	93.14 ± 5.30
Sevoflurane group (%)	95.03 ± 2.89	93.18 ± 5.88	93.65 ± 3.75

*No significant difference was found with respect to accuracy between the two groups.*

### Efficiencies of the Three Networks

Significant differences in the effect scores of the three attentional networks were observed between the two groups and at all three time points using repeated-measures ANOVA.

In the propofol group, patients exhibited significant impairments in all three attentional networks on the 1st postoperative day. The alerting network efficiency recovered to the baseline level, while the orienting network exhibited a delayed recovery on the 5th postoperative day, and no recovery of the executive control network was observed. In the sevoflurane group, significant impairments in all three attentional networks were observed on the 1st postoperative day. Total recoveries were the same for both the alerting and orienting networks on the 5th postoperative day, and the executive control network was still impaired on the 5th postoperative day.

**FIGURE 2:**
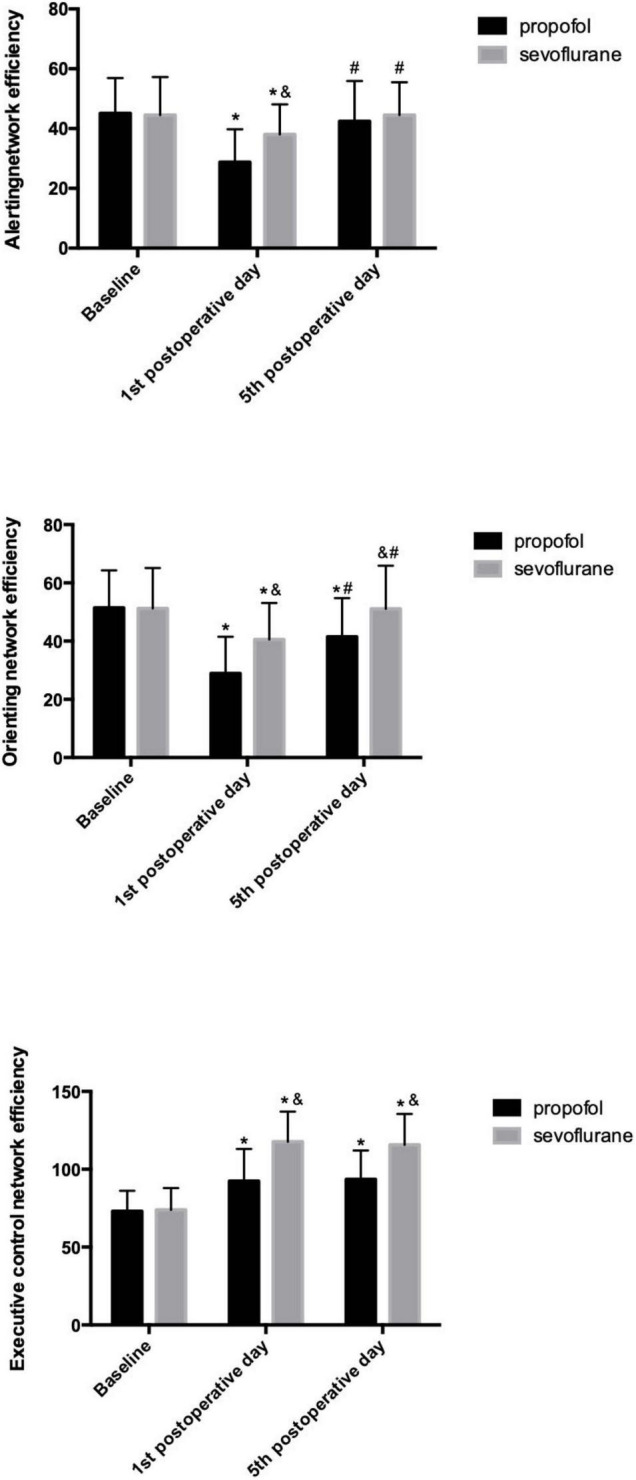
Efficiency of the three attention networks in the propofol and sevoflurane groups. *p < 0.05 versus baseline values; ^#^*p* < 0.05 versus 1st postoperative day; ^&^*p* < 0.05 versus the propofol group.

Pairwise comparisons indicated that compared to the alerting and orienting networks of patients in the sevoflurane group on the 1st postoperative day, those of patients in the propofol group were impaired to a greater extent, while the executive control network was impaired to a lesser extent. On the 5th postoperative day, the alerting networks of both groups recovered to the same level. Patients in the propofol group still showed impairment of the orienting network, while patients in the sevoflurane group recovered to baseline. For the executive control network, patients in the sevoflurane group still exhibited more severe impairment than those in the propofol group. (Table 3 and Figure 2).

**TABLE 3:** Differences at baseline and the 1st and 5th OP sessions between the alerting, orienting and executive networks.

Group	Item	Baseline	1st OP	5th OP
propofol	Alerting	45.1 ± 11.8	28.8 ± 11.0*	42.4 ± 13.5^#^
	Orienting	51.4 ± 12.9	28.9 ± 12.6*	41.5 ± 13.3*^#^
	Executive	73.1 ± 13.1	92.4 ± 20.7*	93.5 ± 18.6*
Sevoflurane	Alerting	44.5 ± 12.7	38.0 ± 10.1^&^*	44.5 ± 11.0^#^
	Orienting	51.2 ± 13.9	40.6 ± 12.5^&^*	51.1 ± 14.8^&#^
	Executive	73.9 ± 14.0	117.7 ± 19.3^&^*	115.7 ± 19.9^&^*

*Data are presented as the mean (SD).*

*p < 0.05 versus baseline values.

*^#^p < 0.05 versus 1st postoperative day.*

*^&^p < 0.05 versus the propofol group.*

## Discussion

Sevoflurane and propofol are frequently used general anaesthetics in clinical practice, and previous studies have shown that anaesthetic agents could be beneficial, deleterious or neutral to the central nervous system (24–27), which indicates that anaesthetics may affect cognitive outcomes after surgery depending on the type of anaesthetic. To understand the effects of different anaesthetics on the three distinct attentional networks, we captured the efficiency of three attention networks in middle-aged women before and after hysterectomy surgery under sevoflurane inhalational anaesthesia or propofol intravenous to explore the impact of different types of anaesthesia on cognitive performance. Significant differences were found in the three attentional networks on the 1st and 5th postoperative days. Compared with patients who received sevoflurane inhalational anaesthesia, those who received propofol intravenous anaesthesia exhibited significantly lower efficiency on the alerting network task and the orienting network task but had fewer difficulties in resolving conflict. The impairment on attention networks after propofol intravenous anaesthesia and the recovery strategies of them were consistent with our previous research (28). ANT was selected as the object of this study because of its sensitivity and applicability for determining slight changes in attention deficits (29). Although we only observed early cognitive dysfunction in this study, we found that the function of the executive control network was significantly impaired and difficult to recover after surgery and anaesthesia. However, the difference in impairment types of the attention network between propofol intravenous anaesthesia and sevoflurane inhalation anaesthesia needs further study.

Propofol intravenous anaesthesia and sevoflurane inhalation anaesthesia are both commonly used in gynaecological surgery, but it is not clear which type of anaesthetic has less influence on cognitive function. A previous study showed that the use of different types of anaesthetics had no significant effect on cognitive function (30). Anaesthetic agents might even have neuroprotective effects against various neural injuries in certain circumstances (17). In contrast, the results of animal studies indicate that conventional standard doses of anaesthetics may lead to long-term learning and memory impairment (31, 32). How general anaesthesia drugs act on the central nervous system, and its pharmacological mechanism is not completely clear, but the current mainstream view holds that the mechanisms of cognitive impairment induced by general anaesthesia may include tau hyperphosphorylation (33), caspase-3 activation (34), β-amyloid deposition (35), increased activity of γ-aminobutyric acid type a receptor in the brain (36) and changes in the central cholinergic transmission of nicotine and muscarinic receptors (37). These processes have all been proven to be directly associated with the development of AD, and a final common pathway may underlie the pathogenesis of AD and PND. The findings of this study point to the phenomenon that propofol and sevoflurane can lead to postoperative cognitive decline.

Although propofol and sevoflurane can both cause postoperative cognitive impairment, the patterns of attention network impairment are different according to our study. The possible reasons may be as follows.

Distinct cognitive and behavioural processes may be regulated by different regions of the brain, and lesions in these areas result in characteristic clinical deficits (38). Formal neuroimaging and functional imaging studies can be used to infer the specific areas of the brain impaired by anaesthesia and surgery. According to previous analyses, the alerting network is responsible for maintaining alertness and warning of stimulation (39), and it is related to the frontal and parietal cortices of the right hemisphere and involves cortical projections of the norepinephrine system (40). The orienting network is responsible for substantial information selection between sensory inputs, affecting the ability to transfer attention cues, and it is related to the superior colliculus, parts of the superior and inferior parietal lobules, frontal eye fields and the temporal parietal junction while also involving the acetylcholine system (41). The executive control network is responsible for cognitive and emotional self-regulation; it is related to frontal areas, including the anterior cingulate cortex, basal ganglia, and lateral prefrontal cortex and is modulated by dopamine (42, 43).

In recent years, with the continuous improvement and development of neuroimaging technology, we have a new understanding of the mechanism of general anaesthesia. At present, functional magnetic resonance imaging (fMRI) and positron emission tomography (PET) are the most widely used in anaesthesia research. According to the changes in local cerebral blood flow (rCBF) and local cerebral glucose metabolism rate (rCMRglu) during anaesthesia, dynamic functional imaging was performed on brain groups to capture the action targets of anaesthetics in the central nervous system. The effects of propofol and sevoflurane on rCBF and rCMRglu are obviously different. Previous studies of propofol found rCBF reductions in the frontal cortex, posterior cingulate, frontal orbital gyrus cortex and cuneiform lobe (44, 45), and studies on the cerebral metabolic rate using positron emission tomography have shown that in the propofol anaesthetised state, the order of the CMR in each brain region from lowest to highest was parietal lobe < occipital lobe < temporal lobe < frontal lobe (46). Previous imaging studies showed that sevoflurane anaesthesia caused a global reduction in rCBF, but the rCBF of the cuneiform lobe, anterior cuneiform lobe and posterior limbic system decreased most obviously (47).

By studying the different changes in the relative cerebral glucose metabolism levels induced by propofol and sevoflurane, it can be inferred that the regions suppressed more by sevoflurane than propofol are mostly distributed in parts of the insula, limbic system, thalamus, pons and cerebellum. In contrast, the regions suppressed more by propofol are mostly distributed in the neocortex of the temporal, parietal and frontal lobes (48). Our results showed that propofol anestheisa had more impairment on alerting and orienting network than sevoflurane anaesthesia while sevoflurane showed more residual impairment of the executive control network which reflected similar patterns with those neuroimaging and functional imaging researches above.

Differences in neurotransmitters may be another reason. Neurotransmitters are actively involved in various brain functions, including movement, attention, emotion, learning, and memory (40, 49). At present, there are many studies on the effects of propofol and sevoflurane on neurotransmitters. Animal experiments have shown that propofol is a GABAA receptor that mainly acts on chloride channel coupling in the brain and has an anaesthetic effect by promoting the release of the inhibitory neurotransmitter gamma aminobutyric acid (GABA) (50, 51). GABAA receptors are also expressed in the locus coeruleus, and GABA neurons in the locus coeruleus have synaptic connections with NE neurons, which can decrease norepinephrine (NE) levels (52). Numerous studies have confirmed that the cholinergic system is an important target of many general anaesthetics. Wang et al. showed that propofol significantly inhibits the release of acetylcholine (53), and Hvarfber et al. showed that propofol exerts anaesthetic effects by inhibiting the reuptake of neurotransmitters such as GABA and dopamine (DA) in central synapses (54). Sevoflurane also has many biochemical actions, and it has been shown to inhibit the function of the nicotinic acetylcholine receptor in the postsynaptic membrane and significantly reduce the transmission of acetylcholine between synapses (55). Nishikawa et al. found that sevoflurane enhanced GABAA receptor-mediated synaptic inhibition in hippocampal interneurons (56), and Silva JH et al. found that sevoflurane increased the levels of extracellular [3H] dopamine in rat brain cortical slices (57). The effects of propofol and sevoflurane on neurotransmitters in the central nervous system are related to the neural mechanism of the attention network, which can reduce the efficiency of the attention network.

In addition to the above reasons, researches about coupling functions (58–60) using EEG (Electroencephalogram) enabled us to unveil a new perspective on how the neurophysiological mechanisms are affected by general anaesthesia. For example, Stankovski et al. found that both propofol and sevofluane increased the alpha–gamma coupling (which plays a prominent role in attention processing (61)) during anaesthesia, but the strength and effect were significantly stronger for sevoflurane (60).

There are also several limitations of the present study. First, this predefined analysis was performed using data from women recruited in only one centre. The generalisability of our results can legitimately be questioned. Second, we lack research on biomarkers in cerebrospinal fluid or serum closely related to cognitive function changes, such as interleukin-6 (IL-6), tumour necrosis factor-α (TNF-α) or S100 calcium binding protein β (S100β). Third, the long-term effects of propofol intravenous anaesthesia and sevoflurane inhalation anaesthesia on cognitive function have also not been studied. Fourth, we did not use neuroimaging examination to confirm the localisation of brain regions with impaired attention networks, such as PET, fMRI and voxel-based morphometry (VBM). The current study provides impetus for further research examining the effects of various anaesthetic agents on postoperative cognitive outcomes.

## Conclusion

Different general anaesthetics can cause different degrees of attention network impairment after surgery. Propofol caused more serious impairment to the patients’ alerting network and orienting network, while sevoflurane caused more serious impairment to the patients’ executive control network. The impairment of the executive control network after the two kinds of anaesthesia could not be restored to the preoperative level in a short time.

## Data Availability Statement

The raw data supporting the conclusions of this article will be made available by the authors, without undue reservation.

## Ethics Statement

The study was approved by the Ethics Committee of Anhui Medical University in Hefei, Anhui, China, in July 2013. (Ethics Committee No. 20130406, Chairperson, Professor Run-ling Wang), and informed consent was obtained from all individual participants. The patients/participants provided their written informed consent to participate in this study.

## Author Contributions

CC: conceptualization, methodology, software, and writing—reviewing and editing. YW: conceptualization, writing—reviewing and editing. JR and WT: validation and software. WW: formal analysis. YL: writing—reviewing and editing. GX: conceptualization, investigation, and software. WZ: funding acquisition, writing—reviewing and editing, and conceptualization. All authors contributed to the article, take responsibility for the integrity of the work as a whole, and approved the submitted version.

## Conflict of Interest

The authors declare that the research was conducted in the absence of any commercial or financial relationships that could be construed as a potential conflict of interest.

## Publisher’s Note

All claims expressed in this article are solely those of the authors and do not necessarily represent those of their affiliated organizations, or those of the publisher, the editors and the reviewers. Any product that may be evaluated in this article, or claim that may be made by its manufacturer, is not guaranteed or endorsed by the publisher.
